# Hidden Circulation and Socio-Sanitary Vulnerability: Rotavirus A and Human Adenovirus Prevalence in Symptomatic and Asymptomatic Children in Central Brazil Post-COVID-19

**DOI:** 10.3390/pathogens14121258

**Published:** 2025-12-09

**Authors:** Jordana Gomes Paulino, Amanda de Oliveira Matos, Yasmin Gomes Passos, Fernanda Franco, Fabiola Souza Fiaccadori, Helioswilton Sales-Campos, Adriana Luchs, Menira Souza, Marcelle Silva-Sales

**Affiliations:** 1 Laboratory of Virology and Cellular Culture, Institute of Tropical Pathology and Public Health, Federal University of Goiás, Rua 235, S/N, Setor Leste Universitário, Goiânia 746050-050, GO, Brazil; jgomes@egresso.ufg.br (J.G.P.); yasminpassos@discente.ufg.br (Y.G.P.); fecfranco@gmail.com (F.F.); fabiola@ufg.br (F.S.F.); menira@ufg.br (M.S.); 2 Laboratory of Mucosal Immunology and Immunoinformatics, Institute of Tropical Pathology and Public Health, Federal University of Goiás, Goiânia 74605-220, GO, Brazil; amandamatos@ufg.br (A.d.O.M.); tonsales@ufg.br (H.S.-C.); 3 Adolfo Lutz Institute, São Paulo 01246, SP, Brazil; driluchs@gmail.com

**Keywords:** Acute Diarrheal Disease, rotavirus A, human adenovirus, viral hidden circulation, socio-sanitary vulnerability

## Abstract

Acute Diarrheal Disease (ADD) remains a major global health concern, with rotavirus A (RVA) and human adenovirus (HAdV) as key viral agents. This study evaluated the positivity rate and molecular characterization of RVA and HAdV in fecal samples from 114 children (0–5 years) in Goiás, Brazil, between January 2022 and December 2023, following the COVID-19 public health emergency. RVA was detected in 13.1% of samples (genotypes G1 and G6 associated with P[8]) and HAdV in 15.8% (genotypes A31, B3, C1, and C6), with two coinfection cases also being detected (1.7%). RVA predominated in unvaccinated 0-to-6-month-old children, while most asymptomatic cases occurred among vaccinated children. HAdV was also mainly identified in this age group, including asymptomatic children. Viral positivity was more common among children without access to treated water. In conclusion, this study underscores the ongoing circulation and genetic diversity of RVA and HAdV among children in Goiás in the post-pandemic period. The findings highlight the influence of vaccination coverage and sanitation on viral positivity, emphasizing the need for integrated public health strategies. Continuous molecular surveillance remains essential to detect emerging genotypes and guide effective prevention and control measures against ADD.

## 1. Introduction

Acute Diarrheal Disease (ADD) is a gastrointestinal inflammatory condition characterized by, at least, three liquid-to-semi-liquid evacuation episodes within 24 h [1,2,3]. It is associated with dehydration, hydro electrolytic disorders and impaired intestinal absorption, thus compromising child development [4,5,6]. It is a global public health issue, with outbreaks recorded in different geographical regions, especially in areas with low socioeconomic levels and poor sanitary conditions [7,8,9,10,11]. Although ADD affects individuals of all age groups, children under five years old have the highest morbidity and mortality rates [12,13]. It is estimated that there are approximately 1.7 billion episodes of infantile ADD, with nearly 444.000 deaths of children under 5 years old annually [14,15]. Considering viral agents, rotavirus A (RVA) and human adenovirus (HAdV) are the most frequent in ADD infantile hospitalizations globally [16,17].

In Brazil, the Acute Diarrheal Disease Monitoring System (MDDA) was implemented in 1994, notifying individual cases of ADD in the Epidemiological Surveillance Information System for Acute Diarrheal Diseases (SIVEP-DDA), where more than 4 million cases and nearly 4000 deaths are recorded annually [18,19]. In Goiás State, Central Brazil, notifications fluctuate yearly, and expanded surveillance for RVA was only implemented in 2007. Although RVA surveillance is ongoing, epidemiological data on HAdV-associated ADD are scarce, and little is known about the socioeconomic and sanitary characteristics of affected populations.

Oral RVA vaccine (VORH) has been implemented in Brazil in March 2006, reducing severity and infant mortality from ADD, especially when combined with measures that provide adequate sanitary conditions [20,21]. However, since 2016, vaccination coverage has been declining, reaching its lowest levels during the COVID-19 public health emergency, and no Brazilian state has reached the RVA vaccination target since then. The combined impact of reduced vaccination rates and the disruption of surveillance activities during the pandemic have likely altered the dynamics of enteric virus circulation in the post-pandemic period [22,23].

Essentially, existing literature is largely based on symptomatic and hospitalized populations, which may underestimate the true prevalence of RVA and HAdV in the community. This study aims to address this gap by investigating RVA and HAdV circulation in both symptomatic and asymptomatic children under five years old in Goiás, a central Brazilian state with variable ADD incidence and persistent vaccination challenges. Additionally, it integrates socioeconomic and sanitary variables, providing a comprehensive view of the factors influencing viral spread.

We hypothesize that assessing both symptomatic and asymptomatic pediatric cohorts will provide a more accurate estimate of RVA and HAdV circulation and will identify socioeconomic and sanitary factors associated with viral positivity.

## 2. Materials and Methods

This is an observational, cross-sectional study designed to detect and molecularly characterize Rotavirus A (RVA) and Human Adenovirus (HAdV) in fecal samples from children in the state of Goiás, Central Brazil. Fecal samples were collected, by convenience, from children aged up to five years old with or without symptoms of ADD, vaccinated or not against RVA—who were admitted to or attended at the pediatric unit of the Hospital das Clínicas of Universidade Federal de Goiás (UFG) between January 2022 and December 2023. The non-probabilistic nature of the sampling reflects the logistical and ethical constraints associated with pediatric sample collection during the post-pandemic period. The sample size (*n* = 114) was defined based on the number of eligible and accessible participants within the study period, providing a descriptive, rather than inferential, epidemiological overview of RVA and HAdV circulation in this population.

Samples were transported at 4 °C to the Laboratory of Virology and Cell Culture (LABVICC) of the Institute of Tropical Pathology and Public Health (IPTSP/UFG). Suspensions (20% in PBS, pH 7.4) were prepared, and viral nucleic acids were extracted using the QIAamp Viral RNA Mini Kit (QIAGEN, Hilden, Germany), following the manufacturer’s instructions. Molecular detection of RVA and HAdV was carried out by RT-qPCR and qPCR assays, respectively, using TaqMan^®^ system targeting NSP3 [24] and Hexon genes [25], respectively. No screening for other enteric microorganisms was performed. Based on the CT value, samples with a CT ≤ 32 were considered suitable for sequencing to ensure there was enough genetic material to generate reliable sequencing results.

A total of 15 samples tested positive for RVA (13.1%) and 18 for HAdV (15.8%). A subset of samples, consisting of 3/15 (20%) RVA-positive and 5/18 (27.8%) HAdV-positive specimens, was successfully sequenced using the Sanger method at the Adolfo Lutz Institute (São Paulo, Brazil). Sequencing was performed using the BigDye^®^ Terminator v3.1 Cycle Sequencing Kit (Applied Biosystems, USA), with primers and reaction conditions previously described in [26] (RVA) and [27] (HadV). Consensus sequences were aligned with reference sequences retrieved from GenBank using BioEdit v7.7.1 [28], and phylogenetic trees were generated with MEGA X v11.0.13 [29], applying the most suitable evolutionary models and a bootstrap score of 2000 replications (values < 70% were excluded). Genotypes were assigned based on phylogenetic clustering with established reference strains rather than by external automated typing tools. Nucleotide sequences were deposited in GenBank under accession numbers PV146220–PV146252.

Socioeconomic and sanitary information was self-reported by parents or guardians through structured interviews and therefore may be subject to recall bias. To minimize inconsistencies, data were cross-checked with available demographic information when possible.

For data analysis, an electronic database was created containing the sociodemographic and laboratory information of the studied population. Initially, the variables underwent a process of cleaning, consistency checking, and standardization to ensure the quality and reliability of the data. Subsequently, the results were tabulated and subjected to chi-square (χ^2^) test using the IBM SPSS Statistics software, version 20. Tables and graphs were constructed from the processed data, removing samples with incomplete patient information from the tabulation, so that the assessment was limited only to the group that completed the questionnaire. Multiple Correspondence Analysis (MCA) was performed based on a Python script (version 3.13), for the analysis of correlation between viral positivity and sanitation factors, vaccination status, sex and housing characteristics (presence of sanitation and street pavement), to provide a more comprehensive analysis of positive cases within the studied group of individuals.

## 3. Results

### 3.1. General Characteristics of the Study Population

Fecal samples were collected from 114 children, 54% (62/114) in 2022 and 46% (52/114) in 2023. Most of the participants, 78.95% (90/114), lived in central Goiás, while 11.4% (13/114) lived in the south, 2.6% (3/114) in the east and 1.75% (2/114) in the northwest. Children who lived in other states but were being treated at HC-UFG accounted for approximately 5.3% (6/114) of the samples collected: one from the state of Bahia, one from Pará, two from Rio de Janeiro and two from São Paulo (Table 1).

The overall positivity rate for RVA was 13.1% (15/114), with 21.0% cases among samples collected in 2022 and 3.8% among those obtained in 2023. For HAdV, the overall positivity rate was 15.8% (18/114), with 14.5% cases detected in samples collected in 2022 and 17.3% in those obtained in 2023. Two patients were positive for both viruses (1.7%). Most positive RVA cases occurred in children residing in Central Goiás, while no RVA detection was recorded in the other mesoregions. One RVA-positive sample was obtained from a RVA-unvaccinated female child, under six months old, from São Paulo State (the economically most relevant area of Brazil), who was admitted to the HC-UFG for treatment of a non-infectious, congenital medical condition. Positive HAdV cases were identified across all mesoregions of Goiás, except the north, with the large majority being detected in Central Goiás. Regarding the coinfection cases, both were from children residing in the center mesoregion, as well (Table 1). Most of the children enrolled in this study were male (57.9%), as were those positive for RVA (61.54%) and the coinfected cases (100%), but many of the HAdV-positive children were female (56.25%).

Regarding age, the highest HadV positivity was observed in the 0–6 months age group (6/16, 37.5%), followed by the 13–24 months group (5/16, 31.25%), 25–60 months group (3/16, 18.75%) and the 7–12 months group (2/16, 12.5%). For RVA, most cases were also detected in children aged 0–6 months (7/13, 53.8%) as well, followed by the 25–60 months group (4/13, 30.8%) and the 13–24 months group (2/13, 15.4%), with no cases being detected in the individuals aged 7–12 months. In the co-infection group, one of the two cases was detected in a child aged 0–6 months, and the other one in a child aged 25–60 months (Table 1). For the school attendance aspect, most of the HAdV- and RVA-positive children did not attend school, while both coinfected cases attended (Table 1).

Although the participants of this study were diverse regarding their socioeconomic conditions, most of the children positive for HAdV or RVA belonged to the group with a family income of 0–1 salary (56.25% and 53.85%, respectively). Regarding infrastructural aspects, many HAdV- and RVA-positive individuals lived in regions with street pavement, treated water and sewage systems, although a lesser percentage of individuals with treated water was found for the infection groups, compared to the overall population percentage (Table 1).

This study also inquired about the RVA-vaccination status of the enrolled individuals, and we found that only 42.1% of the overall children were fully vaccinated. Regarding HAdV cases, an increased percentage of fully vaccinated members were found (68.75%), while for the RVA group, we found that only 38.5% of the cases were fully vaccinated children.

The frequency distribution of non-infected (*n* = 83), HAdV-infected (*n* = 16), RVA-infected (*n* = 13) and co-infected (*n* = 2) groups in the different subcategories of the studied characteristics was assessed by the χ^2^ test, but no significant differences (*p* < 0.05) were found (Table 1).

### 3.2. Symptoms Association to RVA and HAdV Positivity

We also investigated the association of symptoms to the different infectious agents, finding that the majority of the HAdV cases presented two of the four inquired symptoms (diarrhea, vomiting, fever and abdominal pain), with diarrhea and abdominal pain being the most prevalent, followed by fever and vomit, respectively (Table 2). For RVA, most of the cases only showed 1 symptom, with diarrhea being the most prevalent, while vomiting, fever and abdominal pain contributed in an equal manner. For the coinfection cases, one individual was asymptomatic, while the other presented the four symptoms. The number of asymptomatic cases were similar for HAdV and RVA (25% and 23.1%, respectively) (Table 2). Interestingly, two of the three asymptomatic children with RVA, and the asymptomatic coinfected child, had been fully vaccinated against RVA.

### 3.3. Nucleotide Sequencing and Phylogenetic Analysis of RVA-Positive Samples

**Figure 1:**
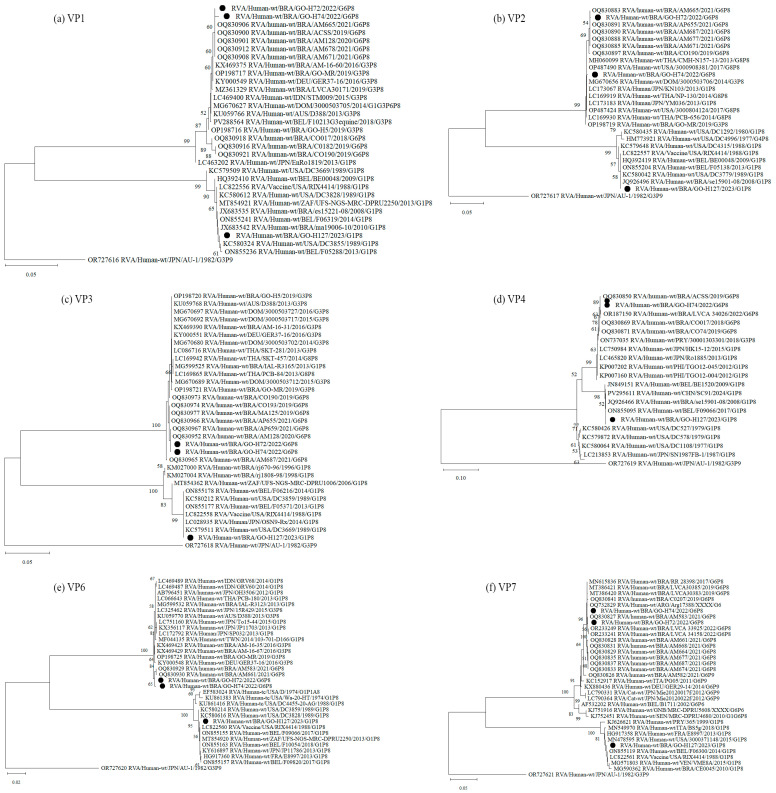
Phylogenetic comparisons of the structural proteins of G6 and G1 study strains, to reference RVA strains from GenBank. The phylogenic trees were constructed in the MEGA X software for VP1 (**a**), VP2 (**b**), VP3 (**c**), VP4 (**d**), VP6 (**e**) and VP7 (**f**) genes. Genotypes lineages are displayed on the right side of each phylogenetic tree, and the study strains are indicated by black dots. Internal nodes were represented by bootstrap values obtained from 2000 replicates and values below 70% were excluded.

**Figure 2:**
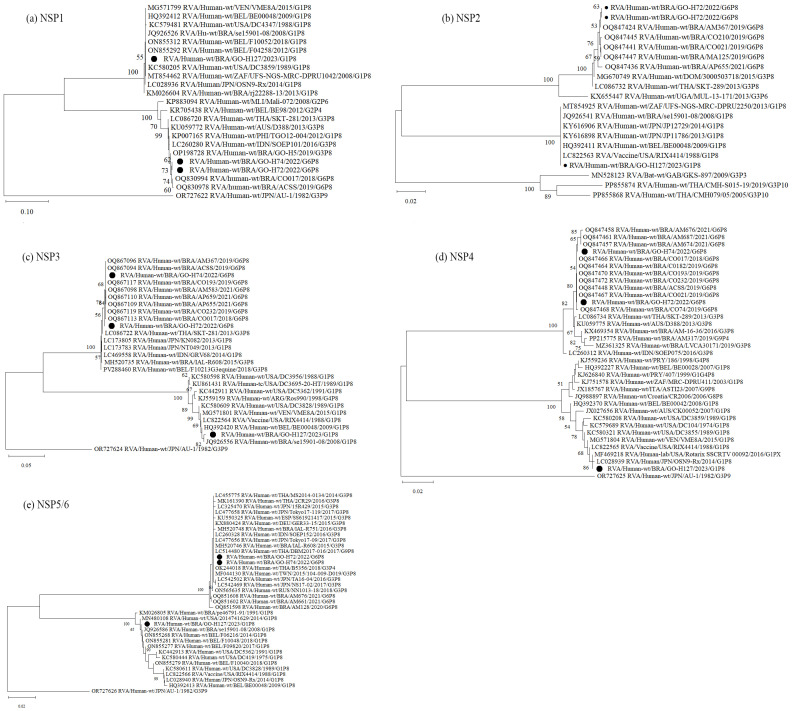
Phylogenetic comparisons of the non-structural proteins of G6 and G1 study strains, to reference RVA strains from GenBank. The phylogenic trees were constructed in the MEGA X software for NSP1 (**a**), NSP2 (**b**), NSP3 (**c**), NSP4 (**d**) and NSP5 (**e**) genes. Genotypes lineages are displayed on the right side of each phylogenetic tree, and the study strains are indicated by black dots. Internal nodes were represented by bootstrap values obtained from 2000 replicates and values below 70% were excluded.

Analysis of the genomic constellation of the sequenced RVA-positive strains showed that two of them are classified as G6-P[8]-3-I2-R2-C2-M2-A2-N2-T2-E2-H2 and one as G1-P[8]-1-I1-R1-C1-M1-A1-N1-T1-E1-H1. Phylogenetic analysis of the 11 RVA genes G6P[8]-3 study strains cluster with other G6, G1 and G3 associated with P[8]-3 strains, all displaying a DS-1-like constellation that has circulated globally over the past decade. The G1P[8]-1 sample was obtained from a diarrheic child under 6 months of age, living in an area without treated sewage and a household income of up to one minimum wage. Phylogenetic analysis of all genes showed clustering with the attenuated VORH strain and VORH-related strains circulating globally. The child had received a single VORH dose less than one month before sample collection, suggesting possible vaccine shedding (Figure 1 and Figure 2).

### 3.4. Nucleotide Sequencing and Phylogenetic Analysis of HAdV-Positive Samples

**Figure 3:**
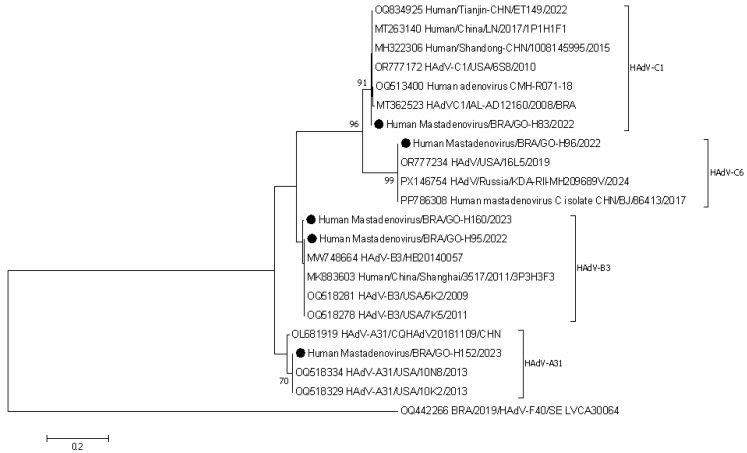
Phylogenetic comparisons of the hexon gene of HAdV-positive study strains, to reference HAdV strains from GenBank. The phylogenic tree was constructed in the MEGA X software. Genotypes are displayed on the right side of phylogenetic tree, and the study strains are indicated by black dots. Internal nodes were represented by bootstrap values obtained from 2000 replicates and values below 70% were excluded.

Molecular analysis of the partial gene sequences encoding the hexon protein revealed the circulation of four distinct HAdV genotypes: A31, B3, C1, and C6. Phylogenetic reconstruction was performed using the Kimura 2-parameter (K2) model to determine the evolutionary relationship among the circulating strains, with the resulting phylogenetic tree presented in Figure 3.

### 3.5. Multiple Correspondence Analysis (MCA) for RVA and HAdV Positivity

**Figure 4:**
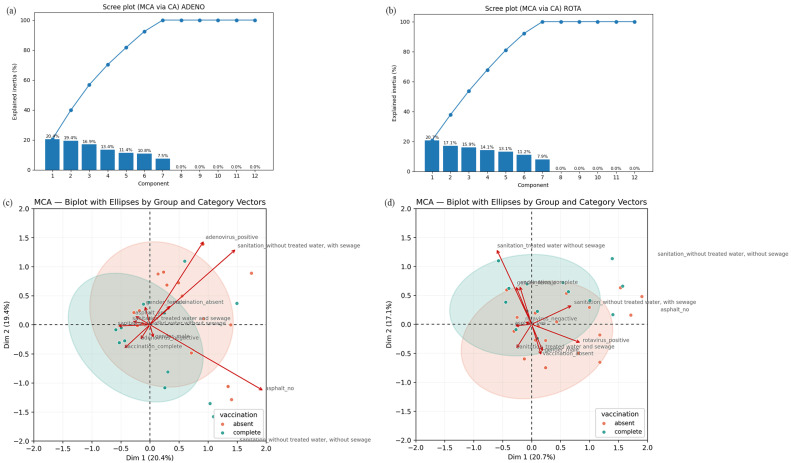
Results of the Multiple Correspondence Analysis (MCA). (**a**,**b**) Screen plot from HAdV (**a**) and RVA (**b**) MCA, showing the percentage of inertia explained by each dimension. Dimensions 1 and 2 concentrate most of the data variability and were used to interpret the association patterns. (**c**,**d**) MCA biplot graphs illustrating the relationship between HAdV (**c**) and RVA (**d**) positivity, vaccination status, and sociosanitary factors in children from Goiás, Brazil. The graphs show the associations among RVA detection status (positive/negative), RVA vaccination status (complete/absent), and selected sociosanitary factors (treated water access, sewage infrastructure and road paving). The first dimension (*x*-axis) accounts for variance, primarily separating individuals based on sanitation access and vaccination status. The second dimension (*y*-axis) reflects RVA positivity. Categories located close to the plot origin have less influence on the axes.

MCA was conducted to assess, in a more dynamic manner, the association of different socioeconomic, sanitary and housing variables to HAdV and RVA positivity. For HAdV positivity, the analysis revealed a strong association between the sanitation category and viral positivity along the first dimension (20.4% of variance explained) (Figure 4a). The second dimension (19.4%) primarily highlights the distinction between gender and vaccination status (Figure 4b). The HAdV-Positive category clusters with indicators of poor infrastructure, specifically absent sewage and lack of treated water. Conversely, the HAdV-Negative category is associated with the complete RVA vaccination status, suggesting that both improved sanitation and high vaccination coverage may contribute to reduced viral circulation (Figure 4b).

For RVA positivity, the first dimension (20.7% of variance explained) primarily captured the relationship between the vaccination status of the children and their access to sanitation (Figure 4c). The second dimension (17.1%) was mainly driven by the RVA positivity index (Figure 4d). Visually, the group with complete RVA vaccination clustered near the category representing treated water access and RVA-negative results, suggesting a strong protective association (Figure 4d). Conversely, the RVA-positive category is associated with absent vaccination status and indicators of poor infrastructure (Figure 4d).

## 4. Discussion

The positivity rates for RVA and HAdV vary geographically and over time, influenced by sanitary and socioeconomic conditions, access to health services, and the particularities of the protocols used for diagnosis and sensitivity of the methodologies applied [20,30]. In Brazil, the implementation of VORH in the National Immunization Program (PNI) and the expansion of sentinel monitoring units have intensified the surveillance of ADD in the country [2,19]. Consequently, there was an initial increase in positivity rates for RVA, followed by a sustained reduction in subsequent years, accompanied by lower hospitalization and a reduction in death rates [31,32,33]. In the initial years following the introduction of the vaccine, there was also an increase in ADD notifications associated with HAdV, suggesting that other gastroenteric viruses could take the epidemiological lead in ADD [34,35]. However, the increased processing of fecal samples after the introduction of the vaccine may have represented a bias, since there was a reduction in the HAdV positivity rate in subsequent years, indicating a possible collective protection provided by VORH [36,37].

Several Brazilian studies have demonstrated a shift in the age group affected by RVA-associated ADD, with a higher frequency between 25 and 48 months [33,38,39,40]. Although the present study found a high positivity rate among children up to 6 months old (53.8%), which had not been reported in recent years, we also found a considerable detection in the 25–60 months age group (30.8%). This new scenario may suggest that the relaxation of social isolation along with a reduction in vaccination coverage, may have favored the occurrence of ADD in children, who had previously been away from social interaction, but who had contact with family members who resumed face-to-face activities. HAdV positivity by age group was consistent with other studies carried out in Brazil [34,35,41], indicating no change in the age group affected after the introduction of VORH.

This study investigated the circulation of RVA and HAdV even in asymptomatic individuals, unlike studies that had focused solely on symptomatic populations. It was observed that the primary objectives of the VORH in reducing the severity of ADD cases are being achieved. However, only 42% (48/114) of the studied children were fully vaccinated against RVA. Since 2016, vaccination coverage has declined in Brazil, with particularly low rates during the COVID-19 pandemic: between 2013 and 2015, VORH coverage levels remained above 90%, dropping to less than 78% in the period of 2020–2022 [42]. In Goiás, specifically, the vaccination coverage followed this same trend: between 2013 and 2015, the coverage was above 96%, reaching over 100% in 2013, while in the 2020–2022 period, the levels varied between 80% and 74% [42. Misinformation about vaccine efficacy, fear of adverse side effects, disregard for immunization against diseases that have already been eradicated, and difficulty in accessing health clinics are possible factors associated with vaccine resistance. Which intensified during the pandemic period, contributing to the sharp decline in vaccination coverage against RVA [43,44].

The spread of RVA and HAdV is usually intensified when associated with places of large agglomeration, such as hospitals, schools and nursery, poor sanitation and lack of hygiene [7,10,45,46]. However, investments that only promote better sanitary conditions are not enough to control the situation, as observed in developed countries that may present children affected by ADD [21,47]. Similarly, encouraging vaccination alone also has limited effectiveness [21,48]. In response to this challenge, in 2015, the United Nations Children’s Fund (UNICEF) and the World Health Organization (WHO) issued a statement highlighting the importance of integrative measures to reduce child mortality from ADD. In this regard, aside from the importance of vaccination, the statement also encouraged breastfeeding, the adoption of good hand hygiene practices, access to treated water and better sanitation conditions [14]. In Brazil, according to the latest census of 2022, 82.9% of the population have access to treated water and 62.5% of the residences had access to sewage collection systems, while in the year 2000, these values were 80.3% and 44.4%, respectively [49]. These values reflect a progress in national sanitation improvements, although the data also show significant regional disparities: while the Brazilian Southeast showed 86.1% of households with sewage collection, Central Brazil had 52.7%, and the North region, 22.8% [49].

The identification of co-infections in this study reinforces the value of multiplex molecular diagnostics in clinical practice, ensuring all causative agents are captured. This evidence, combined with existing data, should inform discussions on refining current vaccination strategies to enhance protection against infantile ADD cases.

Studies conducted in the previous VORH period showed RVA positivity rates ranging from 3.8% to 19% among children with ADD [50,51,52,53]. In Brazil, the post-vaccination scenario, surveillance studies documented heterogeneous but generally declining RVA detection among children with ADD. A laboratory-based, multicentric analysis of 6109 fecal specimens collected across 18 Brazilian states reported RVA positivity of 23.7% in 2006, 16.8% in 2007, 22.9% in 2008 and 18.3% in 2009, documenting early post-vaccine fluctuations and age-specific shifts in detection [33]. Later multicentric Brazilian surveillance studies confirm a lower overall baseline endemicity [40,54]. This sustained reduction suggests the vaccine has effectively lowered the incidence of rotavirus gastroenteritis, yet the persistence of positivity underscores the necessity of continuous molecular and epidemiological monitoring to evaluate long-term vaccine effectiveness and track potential strain evolution.

In the previous VORH period, the G1P[8], G2P[4], G3P[8], G4P[8] and G9P[8] genotypes were the most detected throughout Brazil [27,52,55] and even after the introduction of the vaccine, they continued to circulate with varying periods of re-emergence [26,56,57,58]. Worldwide, in the post-pandemic era, a trend towards a decrease in the prevalence of G3 viruses has been noted, with the emergence of previously uncommon genotypes becoming prevalent, including G9 and G12 RVAs [59,60], although G1P[8], G3P[8] and G2P[4] genotypes are still frequently detected [59,61,62,63]. The G1 and P8 genotypes detected in this study are constituents of the two vaccines licensed in Brazil and account for more than 50% of RVA infections in children [33,54]. The G1-I genotype has re-emerged in Brazil following the relaxation of social isolation measures. The sample H127, referring to a 2-month-old child, was grouped in the same cluster of G1-I prototypes from shedding vaccination, indicating a suggestive case of shedding vaccination, although there is no confirmation on the date of vaccination. Although commonly identified in vaccine-shedding cases, the G1P[8] genotype is sporadically detected worldwide, even in countries where Rotarix is included in the national immunization program. Taken together, these findings reinforce the genetic stability of G1P[8].

The G6 genotype is associated with different P genotypes and has shown restricted and punctual circulation in Brazil. The G6 genotype is commonly associated with animal strains, especially cattle and felines, and have been sporadically detected worldwide since the 1980s [64,65,66,67,68,69]. In recent years, especially in the scenario immediately preceding and during the COVID-19 pandemic, there has been an increase in the circulation of the G6P[8] genotype (G6-I P[8]-3) in different Brazilian states and in other countries as Argentina and Belgium [40,70,71]. The H72 sample, genotyped as G6, was grouped in the same cluster as the G6-I P[8]-3 prototypes detected in Brazil between 2017 and 2022, proving to be distant from the G6 that circulated sporadically in the country in previous decades. A zoonotic transmission of these G3P[8] has been suggested, which was reinforced by the significant similarity between the VP7 gene of RVA strains detected in cats and the human-detected strains from Brazil [40,72]. It has been suggested that the current G6P[8] RVAs were formed by the reassortment between the backbone of the circulating equine-like G3P[8] with a feline-like G6 gene, resulting in a strain that is likely able to evade immune response, and has the potential to replace the equine-like G3P[8], which was predominant until then [40,58,72].

Since the introduction of VORH, the P[8]-3 lineage has predominated in Brazil, occurring in combination with different VP7 genotypes [26,58,73]. Sequence analysis of the VP4 gene also identified the circulation of the P[8]-3 genotype in the present study, suggesting the potential establishment of the G6-IP[8]-3 strain in the country. Ongoing molecular surveillance of RVA strains is essential to track the spread of emerging G6-I P[8]-3 lineages and to anticipate genotypic shifts that may affect the long-term efficacy of VORH globally.

HAdV analysis also show fluctuations in positivity rates over time. Studies conducted in different countries have shown variations in HAdV positivity rates of between 3.9% and 50.2% in children with ADD [35,36,74,75]. Studies have described outbreaks of HAdV-F41 associated with hepatitis after the relaxation of social isolation measures in 2022 [76,77,78]. Although HAdV positivity was observed in the present study, no association was found with reported hepatitis cases. This discrepancy may be related to the challenges of obtaining fecal samples during this period, which could have limited the detection of specific outbreaks. Furthermore, most studies investigating RVA and HAdV circulation have primarily focused on symptomatic individuals, often hospitalized children with ADD. Such selective sampling may partially account for the differences between our findings and those of previous reports, as it tends to exclude asymptomatic infections and may overrepresent more severe clinical presentations. By including both symptomatic and asymptomatic children, the present study provides a broader and more representative understanding of viral circulation dynamics in the post-pandemic context.

HAdV species A, B, and C are primarily implicated in respiratory disease pathogenesis [79,80,81]. However, the role of their genotypes in ADD cases warrants attention. Specifically, HAdV genotypes A31, C1, C6, and B3 have been identified in ADD surveillance efforts globally [82,83,84]. Although the frequency of these genotypes is relatively low compared to the common enteric species HAdV-F40/41, several studies confirm the capacity of A31, B3, C1, and C6 to be shed in the feces of children with diarrhea [85,86,87,88]. The detection of HAdV-A31 in diarrheic stools has been reported more frequently, with records of high genetic stability among circulating strains over time worldwide [89,90,91,92]. Currently, HAdV-A31 represents a significant risk of morbimortality among children undergoing hematopoietic stem cell transplantation (HSCT), with frequent nosocomial outbreaks described in pediatric wards [90,91]. HAdV-B and HAdV-C circulate widely worldwide, and the study samples genotyped as HAdV-B3, HAdV-C1 and HAdV-C6, were grouped with strains from different countries circulating in the last decade. HAdV non-enteric genotypes are detected in children, though their direct role in diarrheic disease remains less clear when compared to enteric types, and data on age-specific distribution are still limited in Brazil. Therefore, current knowledge on the epidemiology of genotypes A31, C1, C6, and B3 remains fragmented, underscoring the need for continuous molecular surveillance and studies with systematic collection of clinical, demographic, and genotypic data.

HAdV MCA results suggest a potential correlation indicating that children with a complete RVA vaccination schedule are more likely to live in areas with access to treated water (even when sewage infrastructure is lacking) and exhibit lower rates of RVA detection. Similarly, the association between HAdV positivity and poorer sanitation quality can be observed, where the HAdV-positive group aligns with regions lacking treated water and sewage systems. As in the RVA analysis, unvaccinated individuals cluster near HAdV-positive cases. Therefore, it can be inferred that the absence of RVA vaccination combined with inadequate sanitation is associated with a higher likelihood of both RVA and HAdV detection, while vaccination correlates with a trend toward viral negativity.

Despite its contributions, this study faced some limitations. First, the samples were obtained by convenience and were limited to children attending a single hospital unit, which may not represent the broader population of Goiás and may introduce a potential selection bias. All data were obtained exclusively through interviews with the child’s parents or guardians, without prior access to the HC-UFG medical records, which may have led to recall bias. Although real-time PCR screening is a highly sensitive method, several samples showed high Ct values, making sequencing and genotyping unfeasible. Furthermore, this study did not include testing for other enteric pathogens such as norovirus or astrovirus, which limits the interpretation of more possible coinfections and the overall etiological profile of ADD. The cross-sectional design also precludes causal inferences, and the absence of multivariate statistical analysis restricted the ability to evaluate more robust associations between viral positivity and socioeconomic or sanitary variables. Despite these limitations, the data contribute valuable insights into the post-pandemic circulation of RVA and HAdV in both symptomatic and asymptomatic children, providing a more accurate overview of ADD in Goiás.

## 5. Conclusions

Investigating the socioeconomic and sanitary conditions of populations affected by ADD, combined with integrative public health measures, is essential for understanding the circulation dynamics of RVA and HAdV and for reducing child morbidity and mortality. In this study, viral positivity was mainly observed in younger children (0–6 months old), and the majority lived in areas with access to treated water and sewage systems. The overall RVA positivity rate was consistent with other studies conducted in the region, while HAdV showed an apparent increase in circulation in Goiás in the post-pandemic period. Molecular analyses revealed the re-emergence of the RVA genotype G1-I and may suggest the potential establishment of genotype G6-I P[8]-III, which could, over time, replace the predominant equine-like G3P[8] circulation in Brazil. The detection of HAdV genotypes A31, B3, C1 and C6—already reported in Goiás but still poorly understood in terms of their association with ADD—highlights the need for further investigation. Taken together, these findings reinforce the importance of continuous molecular and epidemiological surveillance that includes both symptomatic and asymptomatic populations, especially in the context of the post-COVID-19 public health emergency and the recent decline in RVA vaccination coverage in Brazil.

## Figures and Tables

**Table 1:** Sociodemographic, Clinical and Environmental Characteristics of the Study Population and Association with Rotavirus A (RVA) and Human Adenovirus (HAdV) Positivity in Children from Goiás State, Brazil, 2022–2023.

Characteristics	Overall (*n* = 114)	HAdV Positive (*n* = 16)	RVA Positive (*n* = 13)	Coinfection (*n* = 2)	*p*^1^ (*n* = 114)
Age					
0–6 months	56 (49.1%)	6 (37.5%)	7 (53.8%)	1 (50%)	0.76
7–12 months	12 (10.5%)	2 (12.5%)	0 (0%)	0 (0%)	
13–24 months	20 (17.5%)	5 (31.25%)	2 (15.4%)	0 (0%)	
25–60 months	26 (22.8%)	3 (18.75%)	4 (30.8%)	1 (50%)	
Gender					
Male	66 (57.9%)	7 (43.75%)	8 (61.54%)	2 (100%)	0.41
Female	48 (42.1%)	9 (56.25%)	5 (38.46%)	0 (0%)	
Mesoregions					
Center	90 (78.95%)	13 (81.25%)	12 (92.3%)	2 (100%)	0.792
East	3 (2.6%)	1 (6.25%)	0 (0%)	0 (0%)	
Northwest	2 (1.75%)	1 (6.25%)	0 (0%)	0 (0%)	
North	0 (0%)	0	0 (0%)	0 (0%)	
South	13 (11.4%)	1 (6.25%)	0 (0%)	0 (0%)	
Other states	6 (5.3%)	0	1 (7.7%)	0 (0%)	
Family income					
0–1 salary	44 (38.6%)	9 (56.25%)	7 (53.85%)	0 (0%)	0.142
1.1–2 salaries	27 (23.7%)	4 (25%)	1 (7.7%)	0 (0%)	
2.1–3 salaries	11 (9.6%)	0 (0%)	2 (15.4%)	0 (0%)	
3.1–4 salaries	2 (1.8%)	0 (0%)	0 (0%)	0 (0%)	
>4 salaries	12 (10.5%)	2 (12.5%)	0 (0%)	0 (0%)	
N.I.	18 (15.8%)	1 (6.25%)	3 (23.1%)	2 (100%)	
Vaccination					
No	51 (44.73%)	3 (18.75%)	6 (46.1%)	1 (50%)	0.383
Incomplete	15 (13.16%)	2 (12.5%)	2 (15.4%)	0 (0%)	
Complete	48 (42.1%)	11 (68.75%)	5 (38.5%)	1 (50%)	
Pavement					
No	14 (12.3%)	1 (6.25%)	2 (15.4%)	1 (50%)	0.347
Yes	100 (87.7%)	15 (93.75%)	11 (84.6%)	1 (50%)	
Treated water					
No	22 (19.3%)	5 (31.25%)	3 (23.1%)	1 (50%)	0.321
Yes	92 (80.7%)	11 (68.75%)	10 (76.9%)	1 (50%)	
Sewage system					
No	24 (21.1%)	3 (18.75%)	3 (23.1%)	0 (0%)	0.888
Yes	90 (78.9%)	13 (81.25%)	10 (76.9%)	2 (100%)	
School attendance					
No	83 (72.8%)	12 (75%)	10 (76.9%)	0 (0%)	0.194
Yes	25 (21.9%)	4 (25%)	2 (15.4%)	2 (100%)	
N.I.	6 (5.3%)	0 (0%)	1 (7.7%)	0 (0%)	

The table shows the distribution of the overall population (N = 114), and of the HAdV, RVA and coinfection cases separately, across different categories. The frequency distribution for each category in four groups (non-infected, HAdV-infected, RVA-infected and coinfected) was analyzed by the χ^2^ test, with *p*^1^ being the *p*-value obtained in each analysis. N.I.—not informed.

**Table 2:** Association of type and number of symptoms to the different infectious cases.

Characteristics	HAdV Positive (*n* = 16)	RVA Positive (*n* = 13)	Co-Infection (*n* = 2)	*p* ^1^
Number of symptoms				
0	4 (25%)	3 (23.1%)	1 (50%)	0.101
1	1 (6.25%)	4 (30.8%)	0 (0%)	
2	9 (56.25%)	2 (15.4%)	0 (0%)	
3	2 (12.5%)	2 (15.4%)	0 (0%)	
4	0 (0%)	2 (15.4%)	1 (50%)	
Symptoms				
Diarrhea	7	8	1	0.634
Vomiting	5	5	1	0.835
Fever	6	5	1	0.943
Abdominal pain	7	5	1	0.933

The table shows the distribution of the HAdV, RVA and coinfection cases across two different categories. The frequency distribution for each category in three groups (HAdV-infected, RVA-infected and coinfected) was analyzed by the χ^2^ test, with *p*^1^ being the *p*-value obtained in each analysis.

## Data Availability

The data presented in this study are available upon request due to privacy and ethical reasons.

## References

[1] World Gastroenterology Organisation (2012). Acute Diarrhea in Adults and Children: A Global Perspective.

[2] Brasil M.d.S. (2021). Foodborne and Waterborne Diseases Surveillance: Training Manual.

[3] Brasil Ministério da Saúde (2024). Guia de Vigilância em Saúde.

[4] Lee B., Damon C.F., Platts-Mills J.A. (2020). Pediatric acute gastroenteritis associated with adenovirus 40/41 in low-income and middle-income countries. Curr. Opin. Infect. Dis..

[5] Karampatsas K., Osborne L., Seah M.L., Tong C.Y.W., Prendergast A.J. (2018). Clinical characteristics and complications of rotavirus gastroenteritis in children in east London: A retrospective case-control study. PLoS ONE.

[6] Kaiser P., Borte M., Zimmer K.P., Huppertz H.I. (2012). Complications in hospitalized children with acute gastroenteritis caused by rotavirus: A retrospective analysis. Eur. J. Pediatr..

[7] Seo H., Duan Q., Zhang W. (2020). Vaccines against gastroenteritis, current progress and challenges. Gut Microbes.

[8] Ndwandwe D., Runeyi S., Mathebula L., Wiysonge C. (2022). Rotavirus vaccine clinical trials: A cross-sectional analysis of clinical trials registries. Trials.

[9] GBD 2016 Diarrhoeal Disease Collaborators (2018). Estimates of the global, regional, and national morbidity, mortality, and aetiologies of diarrhoea in 195 countries: A systematic analysis for the Global Burden of Disease Study 2016. Lancet Infect. Dis..

[10] Guarino A., Aguilar J., Berkley J., Broekaert I., Vazquez-Frias R., Holtz L., Lo Vecchio A., Meskini T., Moore S., Rivera Medina J.F. (2020). Acute Gastroenteritis in Children of the World: What Needs to Be Done?. J. Pediatr. Gastroenterol. Nutr..

[11] Clark A., van Zandvoort K., Flasche S., Sanderson C., Bines J., Tate J., Parashar U., Jit M. (2019). Efficacy of live oral rotavirus vaccines by duration of follow-up: A meta-regression of randomised controlled trials. Lancet Infect. Dis..

[12] Yandle Z., Coughlan S., Dean J., Hare D., De Gascun C.F. (2021). Indirect impact of rotavirus vaccination on viral causes of acute gastroenteritis in the elderly. J. Clin. Virol..

[13] Moszak M., Szulinska M., Bogdanski P. (2020). You Are What You Eat-The Relationship between Diet, Microbiota, and Metabolic Disorders-A Review. Nutrients.

[14] UNICEF (2024). Diarrhoea Remains a Leading Killer of Young Children, Despite the Availability of a Simple Treatment. https://data.unicef.org/topic/child-health/diarrhoeal-disease/.

[15] World Health Organization (2024). Diarrhoeal Disease. https://www.who.int/news-room/fact-sheets/detail/diarrhoeal-disease.

[16] Banyai K., Estes M.K., Martella V., Parashar U.D. (2018). Viral gastroenteritis. Lancet.

[17] Posovszky C., Buderus S., Classen M., Lawrenz B., Keller K.M., Koletzko S. (2020). Acute Infectious Gastroenteritis in Infancy and Childhood. Dtsch. Ärzteblatt Int..

[18] Brasil. Ministério da Saúde (2024). DATASUS. Informações de Saúde (TABNET): Mortalidade e Morbidade por Doenças Diarreicas Agudas, Brasil, 2000–2024. https://datasus.saude.gov.br/.

[19] Brasil. Ministério da Saúde (2006). SIVEP-DDA—Sistema de Informação de Agravos de Notificação—Doença Diarreica Aguda.

[20] Lestari F.B., Vongpunsawad S., Wanlapakorn N., Poovorawan Y. (2020). Rotavirus infection in children in Southeast Asia 2008–2018: Disease burden, genotype distribution, seasonality, and vaccination. J. Biomed. Sci..

[21] Sadiq A., Bostan N., Yinda K.C., Naseem S., Sattar S. (2018). Rotavirus: Genetics, pathogenesis and vaccine advances. Rev. Med. Virol..

[22] World Health Organization, UNICEF, Gavi Increases in Vaccine-Preventable Disease Outbreaks Threaten Years of Progress, warn WHO, UNICEF, Gavi. 24 April 2025. https://www.who.int/news/item/24-04-2025-increases-in-vaccine-preventable-disease-outbreaks-threaten-years-of-progress--warn-who--unicef--gavi?utm_source=chatgpt.com.

[23] Bigouette J.P., Callaghan A.W., Donadel M., Porter A.M., Rosencrans L., Lickness J.S., Blough S., Li X., Perry R.T., Williams A.J. (2022). Effects of COVID-19 on Vaccine-Preventable Disease Surveillance Systems in the World Health Organization African Region, 2020. Emerg. Infect. Dis..

[24] Zeng S.Q., Halkosalo A., Salminen M., Szakal E.D., Puustinen L., Vesikari T. (2008). One-step quantitative RT-PCR for the detection of rotavirus in acute gastroenteritis. J. Virol. Methods.

[25] Hernroth B.E., Conden-Hansson A.C., Rehnstam-Holm A.S., Girones R., Allard A.K. (2002). Environmental factors influencing human viral pathogens and their potential indicator organisms in the blue mussel, Mytilus edulis: The first Scandinavian report. Appl. Environ. Microbiol..

[26] da Silva M.F.M., Rose T.L., Gomez M.M., Carvalho-Costa F.A., Fialho A.M., de Assis R.M.S., de Andrade J., Volotao E.M., Leite J.P.G. (2015). G1P[8] species A rotavirus over 27 years—Pre- and post-vaccination eras—In Brazil: Full genomic constellation analysis and no evidence for selection pressure by Rotarix^®^ vaccine. Infect. Genet. Evol..

[27] Souza E.V., de Souza Y., Medeiros R.S., de Azevedo L.S., de Queiroz T.G.A., Sanz-Duro R.L., Marinho R., Komninakis S.V., Timenetsky M., Luchs A. (2021). Diversity of enteric and non-enteric human adenovirus strains in Brazil, 2006–2011. Arch. Virol..

[28] Hall T. (1999). BioEdit: A User-Friendly Biological Sequence Alignment Editor and Analysis Program for Windows 95/98/NT. Nucl. Acids Symp. Ser..

[29] Kumar S., Stecher G., Li M., Knyaz C., Tamura K. (2018). MEGA X: Molecular evolutionary genetics analysis across computing platforms. Mol. Biol. Evol..

[30] Sadiq A., Khan J. (2023). Rotavirus in developing countries: Molecular diversity, epidemiological insights, and strategies for effective vaccination. Front. Microbiol..

[31] De Jesus M.C.S., Santos V.S., Storti-Melo L.M., De Souza C.D.F., Barreto I.D.C., Paes M.V.C., Lima P.A.S., Bohland A.K., Berezin E.N., Machado R.L.D. (2020). Impact of a twelve-year rotavirus vaccine program on acute diarrhea mortality and hospitalization in Brazil: 2006–2018. Expert Rev. Vaccines.

[32] do Carmo G.M., Yen C., Cortes J., Siqueira A.A., de Oliveira W.K., Cortez-Escalante J.J., Lopman B., Flannery B., de Oliveira L.H., Carmo E.H. (2011). Decline in diarrhea mortality and admissions after routine childhood rotavirus immunization in Brazil: A time-series analysis. PLoS Med..

[33] Carvalho-Costa F.A., de Assis R.M.S., Fialho A.M., Araujo I.T., Silva M.F., Gomez M.M., Andrade J.S., Rose T.L., Fumian T.M., Volotao E.M. (2019). The evolving epidemiology of rotavirus A infection in Brazil a decade after the introduction of universal vaccination with Rotarix^®^. BMC Pediatr..

[34] Reis T.A., Assis A.S., do Valle D.A., Barletta V.H., de Carvalho I.P., Rose T.L., Portes S.A., Leite J.P., da Rosa e Silva M.L. (2016). The role of human adenoviruses type 41 in acute diarrheal disease in Minas Gerais after rotavirus vaccination. Braz. J. Microbiol..

[35] do Nascimento L.G., Fialho A.M., de Andrade J., de Assis R.M.S., Fumian T.M. (2022). Human enteric adenovirus F40/41 as a major cause of acute gastroenteritis in children in Brazil, 2018 to 2020. Sci. Rep..

[36] Mello M.S., Malta F.C., Fialho A.M., Burlandy F.M., Fumian T.M. (2025). Molecular Epidemiology of Human Adenovirus from Acute Gastroenteritis Cases in Brazil After the COVID-19 Pandemic Period, 2021–2023. Viruses.

[37] Primo D., Pacheco G.T., Timenetsky M., Luchs A. (2018). Surveillance and molecular characterization of human adenovirus in patients with acute gastroenteritis in the era of rotavirus vaccine, Brazil, 2012–2017. J. Clin. Virol..

[38] Dian Z., Sun Y., Zhang G., Xu Y., Fan X., Yang X., Pan Q., Peppelenbosch M., Miao Z. (2021). Rotavirus-related systemic diseases: Clinical manifestation, evidence and pathogenesis. Crit. Rev. Microbiol..

[39] Gomez-Rial J., Rivero-Calle I., Salas A., Martinon-Torres F. (2020). Rotavirus and autoimmunity. J. Infect..

[40] Gutierrez M.B., de Assis R.M.S., Andrade J., Fialho A.M., Fumian T.M. (2023). Rotavirus A during the COVID-19 Pandemic in Brazil, 2020–2022: Emergence of G6P[8] Genotype. Viruses.

[41] Raboni S.M., Damasio G.A., Ferreira C.E., Pereira L.A., Nogueira M.B., Vidal L.R., Cruz C.R., Almeida S.M. (2014). Acute gastroenteritis and enteric viruses in hospitalised children in southern Brazil: Aetiology, seasonality and clinical outcomes. Mem. Inst. Oswaldo Cruz.

[42] DATASUS, Ministério da Saúde (2025). Tabnet: Imunizações—Cobertura. https://datasus.saude.gov.br/informacoes-de-saude-tabnet/.

[43] Oliveira I.S., Cardoso L.S., Ferreira I.G., Alexandre-Silva G.M., Jacob B., Cerni F.A., Monteiro W.M., Zottich U., Pucca M.B. (2022). Anti-vaccination movements in the world and in Brazil. Rev. Soc. Bras. Med. Trop..

[44] Vasconcellos-Silva P.R., Castiel L.D., Griep R.H. (2015). The media-driven risk society, the anti-vaccination movement and risk of autismo. Cienc. Saude Coletiva.

[45] Lo Vecchio A., Conelli M.L., Guarino A. (2021). Infections and Chronic Diarrhea in Children. Pediatr. Infect. Dis. J..

[46] Joshi M.S., Lole K.S., Barve U.S., Salve D.S., Ganorkar N.N., Chavan N.A., Shinde M.S., Gopalkrishna V. (2019). Investigation of a large waterborne acute gastroenteritis outbreak caused by group B rotavirus in Maharashtra state, India. J. Med. Virol..

[47] Troeger C., Khalil I.A., Rao P.C., Cao S., Blacker B.F., Ahmed T., Armah G., Bines J.E., Brewer T.G., Colombara D.V. (2018). Rotavirus Vaccination and the Global Burden of Rotavirus Diarrhea Among Children Younger Than 5 Years. JAMA Pediatr..

[48] Burke R.M., Tate J.E., Parashar U.D. (2021). Global Experience With Rotavirus Vaccines. J. Infect. Dis..

[49] IBGE (2024). Censo Demográfico 2022—Características dos Domicílios: Resultados do Universo.

[50] Waggie Z., Hawkridge A., Hussey G.D. (2010). Review of rotavirus studies in Africa: 1976–2006. J. Infect. Dis..

[51] Modaress S., Rahbarimanesh A.A., Edalat R., Sohrabi A., Modarres S., Gomari H., Motamedirad M., Sayari A.A. (2011). Human rotavirus genotypes detection among hospitalized children, a study in Tehran, Iran. Arch. Iran. Med..

[52] Leite J.P., Carvalho-Costa F.A., Linhares A.C. (2008). Group A rotavirus genotypes and the ongoing Brazilian experience: A review. Mem. Inst. Oswaldo Cruz.

[53] Carvalho-Costa F.A., Volotao Ede M., de Assis R.M., Fialho A.M., de Andrade Jda S., Rocha L.N., Tort L.F., da Silva M.F., Gomez M.M., de Souza P.M. (2011). Laboratory-based rotavirus surveillance during the introduction of a vaccination program, Brazil, 2005–2009. Pediatr. Infect. Dis. J..

[54] Gutierrez M.B., Fialho A.M., Maranhao A.G., Malta F.C., Andrade J., Assis R.M.S., Mouta S., Miagostovich M.P., Leite J.P.G., Machado Fumian T. (2020). Rotavirus A in Brazil: Molecular Epidemiology and Surveillance during 2018–2019. Pathogens.

[55] Banyai K., Laszlo B., Duque J., Steele A.D., Nelson E.A., Gentsch J.R., Parashar U.D. (2012). Systematic review of regional and temporal trends in global rotavirus strain diversity in the pre rotavirus vaccine era: Insights for understanding the impact of rotavirus vaccination programs. Vaccine.

[56] Santos F.S., Sousa Junior E.C., Guerra S.F.S., Lobo P.S., Penha Junior E.T., Lima A.B.F., Vinente C.B.G., Chagas E.H.N., Justino M.C.A., Linhares A.C. (2019). G1P[8] Rotavirus in children with severe diarrhea in the post-vaccine introduction era in Brazil: Evidence of reassortments and structural modifications of the antigenic VP7 and VP4 regions. Infect. Genet. Evol..

[57] Gutierrez M.B., de Figueiredo M.R., Fialho A.M., Cantelli C.P., Miagostovich M.P., Fumian T.M. (2021). Nosocomial acute gastroenteritis outbreak caused by an equine-like G3P[8] DS-1-like rotavirus and GII.4 Sydney[P16] norovirus at a pediatric hospital in Rio de Janeiro, Brazil, 2019. Hum. Vaccines Immunother..

[58] Oliveira Matos A., Araujo M., Paulino J., Franco F.C., Luchs A., Sales-Campos H., Fiaccadori F., Souza M., Silva-Sales M. (2025). Mutations in the main antigenic sites of VP7 and VP8* from G3P[8] rotavirus a strains circulating in Brazil may impact immune evasion to rotavirus vaccination. Braz. J. Microbiol..

[59] Li Y., Wang S., Liang F., Teng S., Wang F. (2024). Prevalence and genetic diversity of rotavirus among children under 5 years of age in China: A meta-analysis. Front. Immunol..

[60] Redda Y.T., Adamu H., Bergholm J., Lindahl J.F., Blomstrom A.L., Berg M., Sisay Tessema T. (2025). Rotavirus A genotype diversity and antigenic profile in Central Ethiopia: Implications for rotarix^®^ vaccine efficacy. Front. Microbiol..

[61] Gonzalez G., Carr M.J., Byrne H., Colgan A., Hare D., Sawa H., De Gascun C.F., Matthijnssens J., Yandle Z. (2025). Complex evolutionary dynamics including reassortment drive genome diversity in human rotavirus species A circulating in Ireland. Infect. Genet. Evol..

[62] Saikia K., Ahmed R., Das B., Paul S., Ray S.K., Chandra Deka R., Borah P.P., Saharia N., Namsa N.D. (2025). Impact of Rotavac Vaccine on Hospital-Based Disease Prevalence and Strain Diversity in India: A Systematic Review and Meta-Analysis. Rev. Med. Virol..

[63] Sangsiriwut K., Thitanuwat B., Saita T., Prasertsopon J., Lerdsamran H., Puthavathana P., Noisumdaeng P. (2025). Molecular Surveillance and Genotypic Distribution of Rotavirus A, Norovirus GI and GII in Bangkok Wastewater Treatment Plants During COVID-19 Phase in 2023, Thailand. Food Environ. Virol..

[64] Banyai K., Gentsch J.R., Griffin D.D., Holmes J.L., Glass R.I., Szucs G. (2003). Genetic variability among serotype G6 human rotaviruses: Identification of a novel lineage isolated in Hungary. J. Med. Virol..

[65] De Grazia S., Martella V., Rotolo V., Bonura F., Matthijnssens J., Banyai K., Ciarlet M., Giammanco G.M. (2011). Molecular characterization of genotype G6 human rotavirus strains detected in Italy from 1986 to 2009. Infect. Genet. Evol..

[66] Gerna G., Sarasini A., Parea M., Arista S., Miranda P., Brussow H., Hoshino Y., Flores J. (1992). Isolation and characterization of two distinct human rotavirus strains with G6 specificity. J. Clin. Microbiol..

[67] Ianiro G., Delogu R., Camilloni B., Lorini C., Ruggeri F.M., Fiore L. (2013). Detection of unusual G6 rotavirus strains in Italian children with diarrhoea during the 2011 surveillance season. J. Med. Virol..

[68] Ndze V.N., Esona M.D., Achidi E.A., Gonsu K.H., Doro R., Marton S., Farkas S., Ngeng M.B., Ngu A.F., Obama-Abena M.T. (2014). Full genome characterization of human Rotavirus A strains isolated in Cameroon, 2010–2011: Diverse combinations of the G and P genes and lack of reassortment of the backbone genes. Infect. Genet. Evol..

[69] Nordgren J., Nitiema L.W., Sharma S., Ouermi D., Traore A.S., Simpore J., Svensson L. (2012). Emergence of unusual G6P[6] rotaviruses in children, Burkina Faso, 2009–2010. Emerg. Infect. Dis..

[70] Simsek C., Bloemen M., Jansen D., Descheemaeker P., Reynders M., Van Ranst M., Matthijnssens J. (2022). Rotavirus vaccine-derived cases in Belgium: Evidence for reversion of attenuating mutations and alternative causes of gastroenteritis. Vaccine.

[71] Degiuseppe J.I., Martelli A., Barrios Mathieur C., Stupka J.A., Argentinean Viral Gastroenteritis Surveillance N. (2023). Genetic diversity of rotavirus A in Argentina during 2019–2022: Detection of G6 strains and insights regarding its dissemination. Arch. Virol..

[72] Fukuda Y., Kusuhara H., Takai-Todaka R., Haga K., Katayama K., Tsugawa T. (2024). Human transmission and outbreaks of feline-like G6 rotavirus revealed with whole-genome analysis of G6P[9] feline rotavirus. J. Med. Virol..

[73] Silva-Sales M., Leal E., Milagres F.A.P., Brustulin R., Morais V.D.S., Marcatti R., Araujo E.L.L., Witkin S.S., Deng X., Sabino E.C. (2020). Genomic constellation of human Rotavirus A strains identified in Northern Brazil: A 6-year follow-up (2010–2016). Rev. Inst. Med. Trop. Sao Paulo.

[74] Moura P.O., Roberto A.F., Hein N., Baldacci E., Vieira S.E., Ejzenberg B., Perrini P., Stewien K.E., Durigon E.L., Mehnert D.U. (2007). Molecular epidemiology of human adenovirus isolated from children hospitalized with acute respiratory infection in Sao Paulo, Brazil. J. Med. Virol..

[75] Portal T.M., Reymao T.K.A., Quindere Neto G.A., Fiuza M., Teixeira D.M., Lima I.C.G., Sousa Junior E.C., Bandeira R.D.S., De Deus D.R., Justino M.C.A. (2019). Detection and genotyping of enteric viruses in hospitalized children with acute gastroenteritis in Belem, Brazil: Occurrence of adenovirus viremia by species F, types 40/41. J. Med. Virol..

[76] Marsh K., Tayler R., Pollock L., Roy K., Lakha F., Ho A., Henderson D., Divala T., Currie S., Yirrell D. (2022). Investigation into cases of hepatitis of unknown aetiology among young children, Scotland, 1 January 2022 to 12 April 2022. Eurosurveillance.

[77] Rabaan A.A., Bakhrebah M.A., Nassar M.S., Natto Z.S., Al Mutair A., Alhumaid S., Aljeldah M., Garout M., Alfouzan W.A., Alshahrani F.S. (2022). Suspected Adenovirus Causing an Emerging HEPATITIS among Children below 10 Years: A Review. Pathogens.

[78] Sallam M., Mahafzah A., Sahin G.O., On Behalf Of Escmid Study Group For Viral H.-E. (2022). Hepatitis of Unknown Origin and Etiology (Acute Non HepA-E Hepatitis) among Children in 2021/2022: Review of the Current Findings. Healthcare.

[79] Benko M., Aoki K., Arnberg N., Davison A.J., Echavarria M., Hess M., Jones M.S., Kajan G.L., Kajon A.E., Mittal S.K. (2022). ICTV Virus Taxonomy Profile: Adenoviridae 2022. J. Gen. Virol..

[80] Radke J.R., Cook J.L. (2023). Human adenovirus lung disease: Outbreaks, models of immune-response-driven acute lung injury and pandemic potential. Curr. Opin. Infect. Dis..

[81] Ruivo A.P., da Cruz Bauermann M., Gregianini T.S., Dos Santos F.M., Godinho F., Baethgen L.F., Machado T.R.M., Martins L.G., Mondini R.P., Port C.N. (2025). Surveillance of respiratory viruses in severe acute respiratory infections in Southern Brazil, 2023–2024. BMC Infect. Dis..

[82] Kachooei A., Ataei-Pirkooh A., Mir-Hosseinian M., Behnezhad F., Eftekhari M., Hoseini-Fakhr S.S., Jalilvand S., Latifi T., Khouy R.A., Shoja Z. (2025). Viral co-infections in pediatric acute gastroenteritis: Epidemiology of rotavirus, norovirus, adenovirus, and astrovirus in Tehran, Iran (2021–2022). BMC Infect. Dis..

[83] Tahmasebi R., Costa A.C.D., Tardy K., Tinker R.J., Milagres F.A.P., Brustulin R., Teles M., Chagas R.T.D., Soares C., Watanabe A.S.A. (2020). Genomic Analyses of Potential Novel Recombinant Human Adenovirus C in Brazil. Viruses.

[84] Wu B.S., Huang Z.M., Weng Y.W., Chen F.Q., Zhang Y.L., Lin W.D., Yu T.T. (2019). Prevalence and Genotypes of Rotavirus A and Human Adenovirus among Hospitalized Children with Acute Gastroenteritis in Fujian, China, 2009–2017. Biomed. Environ. Sci..

[85] Tang X., Hu Y., Zhong X., Xu H. (2022). Molecular Epidemiology of Human Adenovirus, Astrovirus, and Sapovirus Among Outpatient Children With Acute Diarrhea in Chongqing, China, 2017–2019. Front. Pediatr..

[86] Kumthip K., Khamrin P., Ushijima H., Maneekarn N. (2019). Enteric and non-enteric adenoviruses associated with acute gastroenteritis in pediatric patients in Thailand, 2011 to 2017. PLoS ONE.

[87] Gelaw A., Pietsch C., Liebert U.G. (2019). Genetic diversity of human adenovirus and human astrovirus in children with acute gastroenteritis in Northwest Ethiopia. Arch. Virol..

[88] Qiu F.Z., Shen X.X., Li G.X., Zhao L., Chen C., Duan S.X., Guo J.Y., Zhao M.C., Yan T.F., Qi J.J. (2018). Adenovirus associated with acute diarrhea: A case-control study. BMC Infect. Dis..

[89] Gotting J., Baier C., Panagiota V., Maecker-Kolhoff B., Dhingra A., Heim A. (2022). High genetic stability of co-circulating human adenovirus type 31 lineages over 59 years. Virus Evol..

[90] Fattouh R., Stapleton P.J., Eshaghi A., Thomas A.D., Science M.E., Schechter T., Streitenberger L., Hubacek P., Yau Y.C.W., Brown M. (2022). A Prolonged Outbreak of Human Adenovirus A31 (HAdV-A31) Infection on a Pediatric Hematopoietic Stem Cell Transplantation Ward with Whole Genome Sequencing Evidence of International Linkages. J. Clin. Microbiol..

[91] Lu L., Zhong H., Xu M., Su L., Cao L., Jia R., Xu J. (2021). Molecular and epidemiological characterization of human adenovirus and classic human astrovirus in children with acute diarrhea in Shanghai, 2017–2018. BMC Infect. Dis..

[92] Liu L., Qian Y., Zhang Y., Zhao L., Jia L., Dong H. (2016). Epidemiological aspects of rotavirus and adenovirus in hospitalized children with diarrhea: A 5-year survey in Beijing. BMC Infect. Dis..

